# Single Extracellular Vesicle Analysis Performed by Imaging Flow Cytometry and Nanoparticle Tracking Analysis Evaluate the Accuracy of Urinary Extracellular Vesicle Preparation Techniques Differently

**DOI:** 10.3390/ijms222212436

**Published:** 2021-11-18

**Authors:** Marvin Droste, Tobias Tertel, Stefanie Jeruschke, Robin Dittrich, Evangelia Kontopoulou, Bernd Walkenfort, Verena Börger, Peter F. Hoyer, Anja K. Büscher, Basant K. Thakur, Bernd Giebel

**Affiliations:** 1 Department of Pediatrics II (Pediatric Nephrology), University Hospital Essen, University of Duisburg-Essen, 45147 Essen, Germany; marvin.droste@uk-essen.de (M.D.); stefanie.jeruschke@uk-essen.de (S.J.); peter.hoyer@uk-essen.de (P.F.H.); 2 Institute for Transfusion Medicine, University Hospital Essen, University of Duisburg-Essen, 45147 Essen, Germany; tobias.tertel@uk-essen.de (T.T.); robin.dittrich@uk-essen.de (R.D.); verena.boerger@uk-essen.de (V.B.); 3 Department of Pediatrics III (Pediatric Hematology & Oncology), University Hospital Essen, University of Duisburg-Essen, 45147 Essen, Germany; kontopoulou.evi@gmail.com; 4 Electron Microscopy Unit, Imaging Center, University Hospital Essen, University of Duisburg-Essen, 45147 Essen, Germany; bernd.walkenfort@uk-essen.de

**Keywords:** imaging flow cytometry, extracellular vesicles, urine, extracellular vesicle isolation methods, exosomes, nanoparticle tracking analysis

## Abstract

Small extracellular vesicles isolated from urine (uEVs) are increasingly recognized as potential biomarkers. Meanwhile, different uEV preparation strategies exist. Conventionally, the performance of EV preparation methods is evaluated by single particle quantification, Western blot, and electron microscopy. Recently, we introduced imaging flow cytometry (IFCM) as a next-generation single EV analysis technology. Here, we analyzed uEV samples obtained with different preparation procedures using nanoparticle tracking analysis (NTA), semiquantitative Western blot, and IFCM. IFCM analyses demonstrated that urine contains a predominant CD9^+^ sEV population, which exceeds CD63^+^ and CD81^+^ sEV populations. Furthermore, we demonstrated that the storage temperature of urine samples negatively affects the recovery of CD9^+^ sEVs. Although overall reduced, the highest CD9^+^ sEV recovery was obtained from urine samples stored at −80 °C and the lowest from those stored at −20 °C. Upon comparing the yield of the different uEV preparations, incongruencies between NTA and IFCM data became apparent. Results obtained by both NTA and IFCM were consistent with Western blot analyses for EV marker proteins; however, NTA results correlated with the amount of the impurity marker uromodulin. Despite demonstrating that the combination of ultrafiltration and size exclusion chromatography appears as a reliable uEV preparation technique, our data challenge the soundness of traditional NTA for the evaluation of different EV preparation methods.

## 1. Introduction

Small extracellular vesicles (sEVs) are membrane-coated particles containing nucleic acids, proteins, and lipids of cellular origin [1]. Due to their cell type-specific assembly, they have been qualified as biomarkers for various diseases [2]. sEVs can be detected in all biological fluids, including urine [3]. As the collection of urine is non-invasive and enables acquisition of large sample volumes, small urinary EVs (uEVs) provide an easily accessible source for the identification of novel biomarkers [4,5,6]. However, the applied uEV preparation technique largely influences the yield and purity of uEVs and, thus, the validity of associated biomarkers. Although the International Society for Extracellular Vesicles (ISEV) urges researchers to proceed towards more standardized EV isolation protocols [7], the variety of techniques applied is large. Differential centrifugation procedures including ultracentrifugation (UC) were the gold standard for sEV preparation for years [8]. However, as it has been recognized that UC can promote EV aggregation, does not achieve sufficient purity, and may result in insufficient EV enrichment [9,10], alternative methods are increasingly used for sEV preparation, including polymer-based precipitation, size exclusion chromatography (SEC), and filtration-based methods [11,12]. Concerning the preparation of uEVs, various challenges need to be considered. In addition to the varying concentration of void urine and interindividual differences in uEV profiles [13], especially the presence of highly abundant proteins, such as uromodulin (Tamm Horsfall protein, UMOD), hampers the purification of uEVs [14,15,16,17]. Although several groups already compared different uEV enrichment methods and have also focused on the reduction of co-prepared proteins, no consensus has been reached to date [18,19,20,21,22,23]. The recently published position paper from the ISEV urinary EV task force has outlined that more research is needed to assess strengths and pitfalls of uEV separation protocols, especially as new EV detection technologies may change previous paradigms and offer new opportunities regarding single EV analysis [24].

Critical parameters in evaluating the accuracy of EV preparation methods depend on the analysis technologies used for the characterization of obtained EV samples. Currently, the validity of EV preparation methods is frequently judged by particle quantification technologies, e.g., by nanoparticle tracking analysis (NTA), which we and others introduced in 2011 as an “exosome” quantification method [25,26]. However, upon comparing NTA with imaging flow cytometry (IFCM) analysis, the latter allowing single EV analyses even in non-processed EV-containing samples [27,28,29], it appears that—depending on the initial sample material and the applied preparation method—obtained samples contain far more particles than EVs. Indeed, protein aggregates that are formed in urine appear in NTA as small particles that are indistinguishable from uEVs [30]. Consequently, it needs to be considered that results from NTA and other particle quantification devices in their original design can be misleading, especially if the accuracy and efficacy of EV preparation methods is compared.

Being interested in qualifying EVs as novel urine-derived biomarkers, we decided to re-assess different uEV preparation methods. Now, in addition to the classical analysis techniques, i.e., NTA, Western blot (WB), and transmission electron microscopy (TEM), we performed IFCM analyses for the detection and semi-quantification of single uEVs. To this end, we first identified appropriate antibodies allowing the detection of a vast proportion of uEVs in fresh void urine. For practical reasons, it is often required to work with stored void urine samples. Consequently, we also studied the impact of different storage temperatures on the recovery of antibody-labeled uEVs. Within the method comparison, we focused on the recovery and purity of uEVs that were successfully labeled with the selected antibody. According to the results of the IFCM analyses, other uEV preparation techniques appeared more favorable than those that would have been selected according to the results of the NTA analyses. Since obtained IFCM data are supported by the results of the WB and TEM, but to a lesser extent by the NTA data, we consider IFCM analyses as more specific for the evaluation of EV preparation protocols than conventional NTA analyses or analyses with other conventional particle quantification devices.

## 2. Results

### 2.1. CD9 Is Abundantly Present on Small Urinary EVs of Healthy Donors

**Figure 1:**
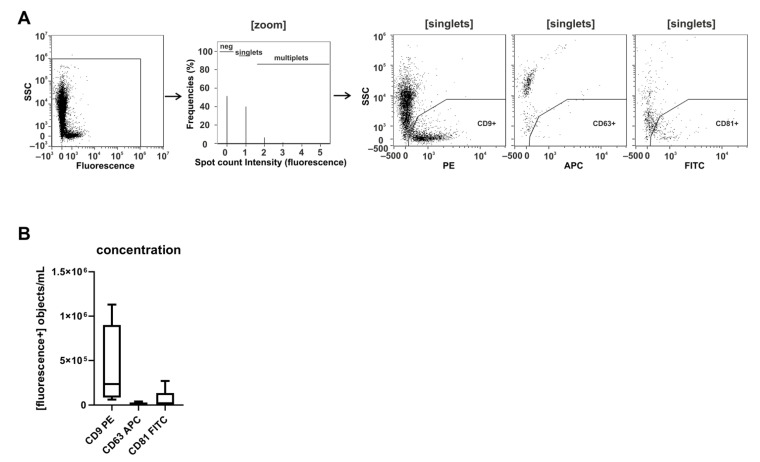
CD9 is abundantly present on small urinary EVs of healthy donors. Freshly pre-processed void urine samples (n = 4) were stained with anti-CD9, anti-CD63, and anti-CD81 antibodies. After a 1-hour incubation step, samples were analyzed using imaging flow cytometry (IFCM). (**A**) Applied gating strategy: from all recorded signals (first plot), data points lacking any spot count signal or showing coincidences were neglected (second plot). Side scatter intensities of single objects are plotted against the fluorescence intensities of CD9-, CD63-, and CD81-labeled objects. (**B**) Box plots reflecting the numbers of recorded CD9^+^, CD63^+,^ and CD81^+^ objects from the different experiments.

Recently, we set up protocols to label sEVs with fluorescent conjugated anti-CD9, anti-CD63, and anti-CD81 antibodies in otherwise non-processed cell culture supernatants, allowing us to immediately analyze the labeled EVs by IFCM [27,29]. Being interested in uEVs in the context of biomarker research, we explored whether a comparable labeling technology could be used for the detection of uEVs in fresh void urine samples of healthy donors (Figure 1). Applying the established protocols, we recovered discrete CD9^+^ uEV populations in all urine samples tested (*n* = 4). In contrast, CD63^+^ and CD81^+^ uEV populations were hardly detectable (CD9: 4.1 × 10^5^ ± 4.8 × 10^5^ objects/mL; CD63: 1.8 × 10^4^ ± 1.4 × 10^4^ objects/mL; CD81: 6.8 × 10^4^ ± 1.0 × 10^5^ objects/mL). Thus, CD9, but neither CD63 nor CD81, presents an abundant uEV marker in fresh human void urine.

### 2.2. Storage Temperature Affects the Recovery of Urinary sEVs

**Figure 2:**
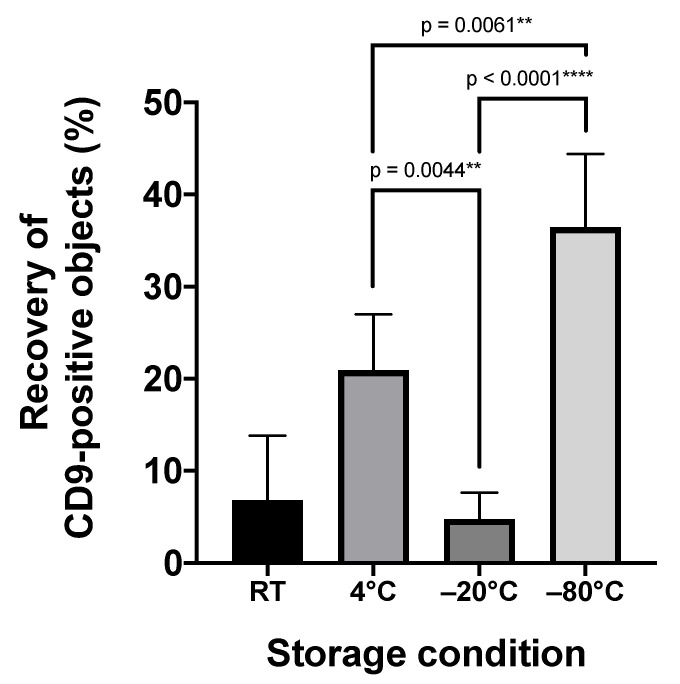
Recovery rates of CD9^+^ uEVs in void urine samples depend on the storage temperature. Freshly prepared, cell-free void urine samples (n = 5) were analyzed by IFCM immediately after antibody staining with anti-CD9 antibodies (no storage) or after storage for 1 month either at room temperature, ((RT) 20 °C), 4 °C, −20 °C, or −80 °C. The recovery of CD9^+^ objects in stored samples was calculated as the ratio of CD9^+^ objects before and after storage. ** *p* ≤ 0.01, **** *p* ≤ 0.0001.

Commonly, biomarker screening projects depend on preserved donor samples. Regularly, urine samples of healthy donors and patients are cryopreserved either at −20 °C or at −80 °C. To test whether cryopreservation affects the quality of respective samples, we evaluated the impact of the storage temperature on the recovery of CD9^+^ uEVs (Figure 2). To this end, five fresh void urine samples were obtained from healthy donors and processed by low-speed centrifugation. Aliquots of these samples were either stored for 1 month at room temperature, 4 °C, −20 °C, or −80 °C, or analyzed immediately, respectively. For uEV marker analyses, stored as well as fresh samples were filtered through 0.22-µm polyether sulfone membrane filters and stained with anti-CD9 antibodies. Stained samples were analyzed by IFCM. Notably, compared to the freshly prepared urine sample, the number of CD9^+^ uEVs declined under all storage conditions, with the lowest decline in samples that had been stored at −80 °C (mean recovery: 36.5% ± 8.0%; mean decline vs. no storage: 4.3 × 10^5^ CD9^+^ objects/mL; *p* = 0.0001 ***) (Figure 2). The highest decline was observed in samples stored at −20 °C (mean recovery: 4.8% ± 2.9%; mean decline vs. no storage: 6.5 × 10^5^ CD9^+^ objects/mL; *p* < 0.0001 ****) (Figure 2). The difference between the mean recoveries of uEVs stored at −20 °C vs. −80 °C was statistically significant (*p* < 0.0001 ****) (Figure 2). Thus, if urine samples cannot be immediately processed for uEV analyses, our results indicate that they are ideally stored at −80 °C.

### 2.3. Imaging Flow Cytometry and NTA Analyses Provide Incongruent Results Regarding the Efficacy of uEV Preparation Methods

**Figure 3:**
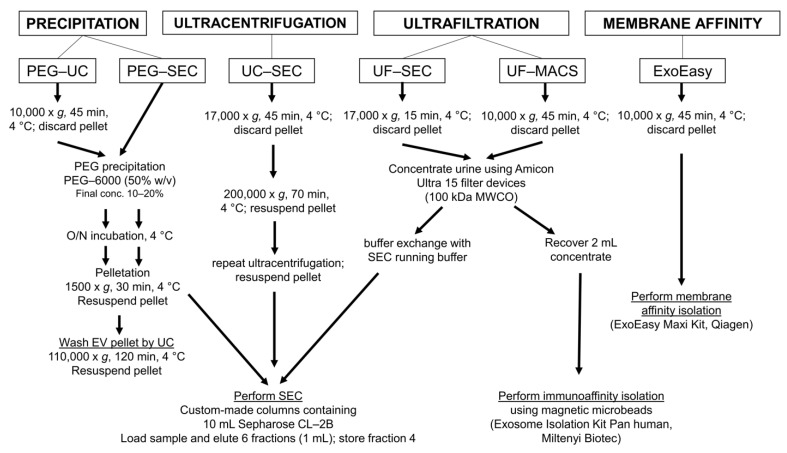
Experimental design of the method comparison using five different methods for uEV preparation and a sixth method for one of the void urine samples. According to published protocols, void urine samples were differently pre-processed, either at 10,000× *g* or at 17,000× *g*. All details for the next procedures are provided. Following uEV preparation, uEV samples were analyzed by IFCM, NTA, Western blot, and transmission electron microscopy. Methods were: PEG precipitation followed by ultracentrifugation (PEG-UC) [31], PEG precipitation followed by size exclusion chromatography (PEG-SEC) [19], ultracentrifugation followed by size exclusion chromatography (UC-SEC) [15], ultrafiltration followed by size exclusion chromatography UF-SEC [32], and the commercial ExoEasy Maxi Kit (Qiagen), which is based on membrane affinity (ExoEasy). The sixth method was ultrafiltration followed by immunoaffinity capturing with commercial anti-CD9, anti-CD63, and anti-CD81 antibody-conjugated magnetic beads (UF-MACS [33]; it was only used for the preparation of EVs from one of the void urine samples.

To evaluate the suitability of different uEV preparation methods, we used urine samples that had been stored at −80 °C. Five independent void urine samples were processed, each with five different protocols frequently applied to enrich uEVs: (1) polyethylene glycol-precipitation followed by ultracentrifugation (PEG-UC) [31] or (2) size exclusion chromatography (PEG-SEC) [19]; (2) UC followed by SEC (UC-SEC) [15]; (4) ultrafiltration followed by SEC (UF-SEC) [32]; and (5) by a one-step protocol using the commercial ExoEasy Maxi Kit based on membrane affinity (Figure 3). The first void urine sample was also processed with a sixth method, ultrafiltration followed by immunoaffinity capturing (UF-MACS) [33]. Due to the presence of EV–microbead aggregates, this sample could neither be analyzed by IFCM nor by NTA. Consequently, MACS preparations were not performed for the remaining four void urine samples.

**Figure 4:**
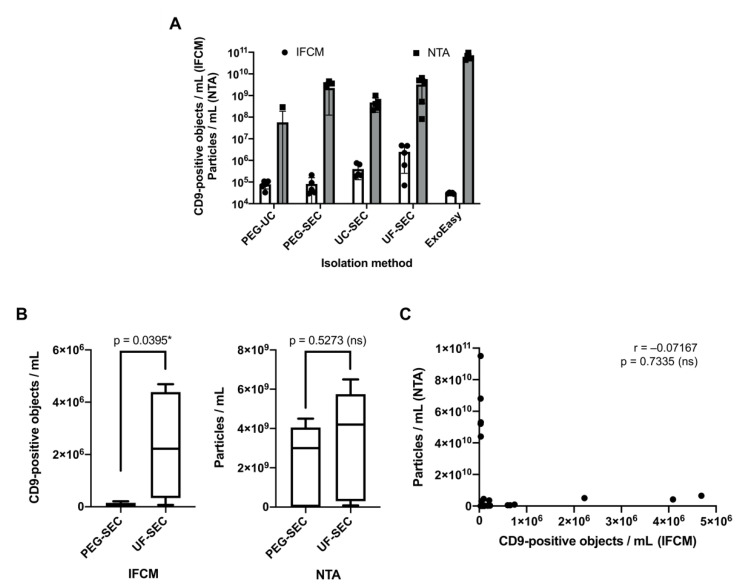
CD9^+^ object recovery rates evaluated by IFCM are incongruent to particle recovery rates evaluated by NTA. Following processing of five void urine samples with the methods shown in Figure 3, obtained samples were analyzed for the presence of CD9^+^ objects by IFCM and for the presence of particles by NTA. (**A**) Side-by-side comparison of average numbers of CD9^+^ objects detected by IFCM and average particle numbers as quantified by NTA. (**B**) Side-by-side comparison of average CD9^+^ object numbers (left) and average particle numbers (right) detected in UF-SEC and PEG-SEC samples (same data as shown in (**A**)). Statistical analysis was performed using Student’s *t*-test. (**C**) Incongruencies of IFCM and NTA measurements as demonstrated by Spearman’s correlation analysis of all recorded IFCM and NTA data (n = 25). * *p* ≤ 0.05.

Following uEV enrichment, samples were at first analyzed for their CD9^+^ uEV content by IFCM and then for total particles by NTA. IFCM analyses of the five different samples with the five preparation protocols revealed the highest CD9^+^ object recovery by the UF-SEC method (2.5 × 10^6^ ± 2.2 × 10^6^ objects/mL), followed by UC-SEC (4.0 × 10^5^ ± 2.7 × 10^5^ objects/mL; UF-SEC vs. UC-SEC: *p* = 0.0278 *). The number of the detected CD9^+^ objects of PEG-SEC and PEG-UC preparations appeared much smaller (PEG-SEC: 8.2 × 10^4^ ± 7.6 × 10^4^ objects/mL; PEG-UC: 7.9 × 10^4^ ± 3.1 × 10^4^ objects/mL; PEG-SEC vs. UF-SEC: *p* = 0.0094 **; PEG-UC vs. UF-SEC: *p* = 0.0093 **); ExoEasy-derived CD9^+^ objects were barely detectable (3.0 × 10^4^ ± 2.0 × 10^3^ objects/mL) (Figure 4A, Supplementary Figure S1).

All samples were also analyzed by NTA. Without reaching statistical significance, ExoEasy preparations revealed the highest particle concentrations (6.2 × 10^10^ ± 2.0 × 10^10^ particles/mL), followed by those prepared with UF-SEC (3.3 × 10^9^ ± 2.8 × 10^9^ particles/mL) and PEG-SEC (2.2 × 10^9^ ± 2.1 × 10^9^ particles/mL). Lower particle concentrations were recorded in UC-SEC (4.8 × 10^8^ ± 3.1 × 10^8^ particles/mL) and PEG-UC (5.8 × 10^7^ ± 1.3 × 10^8^ particles/mL) samples (Figure 4A; individual measurement results with standard deviations for each sample are shown in Supplementary Figure S2).

Thus, beyond the anticipated finding that particles are more abundant than CD9^+^ objects, the results from NTA and IFCM analyses are incongruent to each other. The results of IFCM would favor the UF-SEC method, while, according to NTA analysis, the highest particle yield was obtained with the ExoEasy protocol. The difference is highlighted upon comparing the NTA and IFCM data of the UF-SEC and PEG-SEC samples. While according to the NTA data no significant difference was documented (*p* = 0.5273, Figure 4B), IFCM detected significantly lower CD9^+^ object numbers in PEG-SEC than in UF-SEC samples (*p* = 0.0395 *). Notably, IFCM and NTA data of all analyzed samples showed no significant correlation (r = −0.07167, *p* = 0.7335), demonstrating that the results obtained with the two analysis methods are indeed not congruent. Thus, the choice of the most appropriately appearing uEV preparation method largely depends on the chosen analysis method (Figure 4C).

### 2.4. Correlation Analysis of Western Blot, NTA, and IFCM Results Demonstrate Superior Specificity of IFCM-Based EV Detection

**Figure 5:**
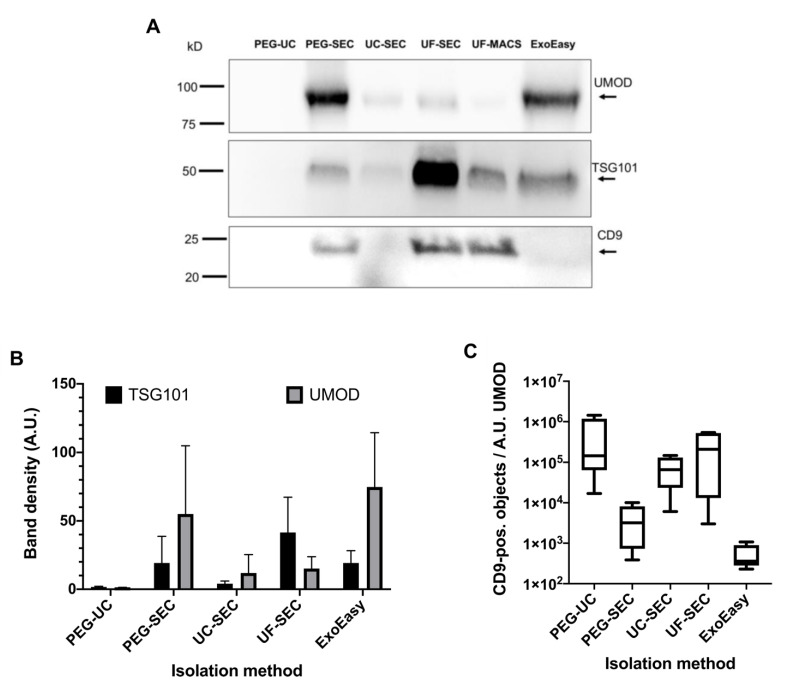
The UF-SEC method outcompetes other techniques in terms of EV protein marker recovery and purity. (**A**) Western blot results of one of the processed void urine samples performed on freshly obtained uEV samples. Sample loading was adjusted to volume equivalents of the initial void urine sample. The uEV samples were separated under reducing and non-reducing conditions. The Western blot performed under reducing conditions was probed with anti-TSG101 and anti-UMOD antibodies. The Western blot performed under non-reducing conditions was probed with anti-CD9 antibodies. Bands were visualized after counterstaining with HRP-conjugated secondary antibodies and addition of chemiluminescent HRP substrate. (**B**) Comparison of average TSG101 and UMOD band intensities of all uEV samples arranged according to the applied purification method. All samples were stored at −80 °C before Western blot analysis, images of all relevant Western blots are shown in Supplementary Figure S3. Error bars indicate the standard deviation. (**C**) Average sample purities are calculated as the ratio of CD9-positive objects to the average UMOD band intensities.

To comply with the MISEV2018 criteria [7] and to potentially solve the inconsistency among the IFCM and NTA data, Western blot (WB) analyses were performed, initially with freshly prepared EV samples obtained from the first void urine sample investigated, which yielded comparably high CD9^+^ object and particle amounts. These EV preparations, including the aforementioned UF-MACS sample, were separated under reducing and non-reducing conditions. The membrane derived from the reducing WB was sequentially probed with anti-uromodulin (UMOD) and anti-TSG101 antibodies, whereas the membrane derived from the non-reducing WB was probed with anti-CD9 antibodies (Figure 5A). UMOD, an abundant contaminating urinary protein, was detected in high amounts in PEG-SEC and ExoEasy samples and lower amounts in UC-SEC and UF-SEC samples. Hardly any UMOD was recovered in the UF-MACS sample. Unexpectedly, no bands, either for UMOD or for TSG101 and CD9, were detected in the PEG-UC preparation. By far, the highest TSG101 content was obtained by the UF-SEC method, followed by ExoEasy, UF-MACS, and PEG-SEC. Hardly any TSG101 was recovered applying UC-SEC. UF-SEC and UF-MACS contained comparable amounts of CD9, which were higher than in the PEG-SEC sample. No CD9 was recovered in UC-SEC and ExoEasy samples (Figure 5A).

To substantiate the data, reducing and non-reducing WBs were performed on all obtained EV samples in parallel, including the samples that had initially been tested in WB. CD9 was not detected in any of the samples, all of which had been stored at −80 °C before, not even in the uEV samples of the first void urine, which had shown CD9 bands in the initial WB from freshly prepared EVs. Since CD9 was well detected in these WBs in the control lanes, the results of the WBs are trustable, supporting unpublished discussions in the field that storage of uEV samples can affect the WB detectability of certain marker proteins, including CD9.

In contrast, the reducing WBs showed clear TSG101 und UMOD bands. The band intensities of the uEV samples from the first processed void urine were comparable in the WB before and after uEV storage. Consequently, we focused on TSG101 and UMOD WB data in subsequent analyses (Supplementary Figure S3).

The intensities of the TSG101 bands were quantified by densitometry (Figure 5B). Notably, the strongest TSG101 bands were recovered from the UF-SEC method throughout all samples tested, thus supporting UF-SEC as a method of choice for high uEV recovery, in line with the previously acquired IFCM data. Of note, neither TSG101 nor UMOD was recovered in two PEG-prepared samples, irrespective of whether they were subsequently processed by SEC or UC (HD4, HD5, Supplementary Figure S3). However, in those two samples, TSG101 was also recovered using UC-SEC, albeit in lower concentrations than in the UF-SEC and ExoEasy samples. The UMOD concentration in all tested samples was lower in UF-SEC samples than in the ExoEasy samples. Thus, the ratio between the intensity of the TSG101 to the UMOD band was highest in UF-SEC preparations (due to the absence of TSG101 bands, no such ratios were calculated for PEG processed samples) (Figure 5C). Overall, our results demonstrated that UF-SEC achieved superior uEV yields with increased purity compared to the other methods tested here.

**Figure 6:**
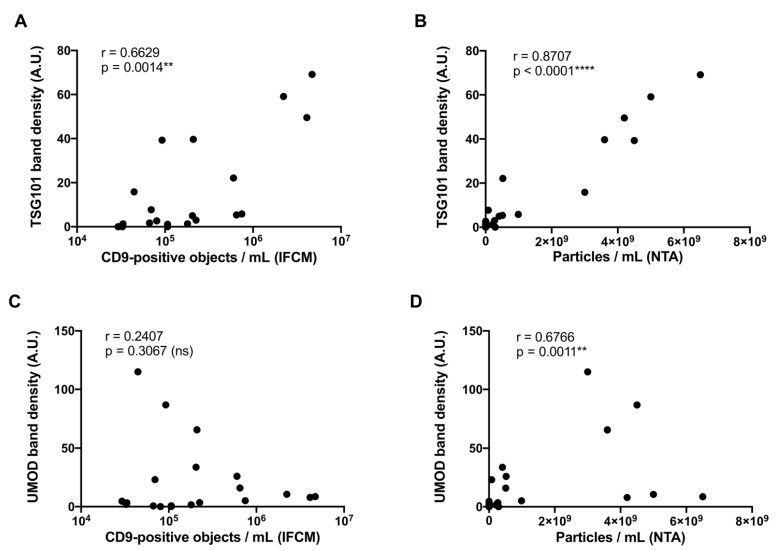
IFCM but not traditional NTA specifically detects EVs for the evaluation of EV preparation methods. Correlation analyses of acquired Western blot band intensities (TSG101 and UMOD) with CD9^+^ object numbers as determined by IFCM or with average particle numbers as determined by NTA (n = 20) applying Spearman’s correlation coefficient analysis. (**A**) Correlation analysis of CD9^+^ objects and TSG101 band intensities. (**B**) Correlation analysis of particle numbers and TSG101 band intensities. (**C**) Correlation analysis of CD9^+^ objects and UMOD band intensities. (**D**) Correlation analysis of particle numbers and UMOD band intensities. ** *p* ≤ 0.01, **** *p* ≤ 0.0001.

Next, we correlated the protein concentration of TSG101 and UMOD derived from the densitometric WB analyses with corresponding IFCM and NTA data. Data from ExoEasy samples were not included in this analysis, as IFCM measurements based on CD9 were below the detection limit. Of note, MACS- and ExoEasy-derived preparations contained similar amounts of TSG101, yet CD9 was only detected in the MACS samples. Therefore, ExoEasy seems to enrich a tetraspanin-negative fraction of particles, as previously reported in a study using plasma [34]. A moderate positive correlation between TSG101 band density and the numbers of CD9^+^ objects measured by IFCM as well as a strong positive correlation between particle numbers measured by NTA and TSG101 band densities were found (IFCM: r = 0.6629, *p* = 0.0014 **, Figure 6A; NTA: r = 0.8707, *p* < 0.0001 ****; Figure 6B). Notably, a moderate positive correlation among the intensities of UMOD bands and particle numbers measured by NTA was also obtained (r = 0.6766, *p* = 0.0011 **; Figure 6D), while no statistically significant correlation was recognized among the UMOD concentration and the number of CD9^+^ objects measured by IFCM (r = 0.2407, *p* = 0.3067 (ns); Figure 6C). Thus, our data indicated that NTA-based EV enumeration is affected by impurities, while impurities apparently do not affect results of IFCM. Consequently, IFCM analyses enable enhanced specificity towards EVs compared to traditional NTA and largely improve the accurate estimation of EV vs. contamination abundancy.

### 2.5. Electron Microscopy Supports UF-SEC as a Suitable Method for uEV Preparation

**Figure 7:**
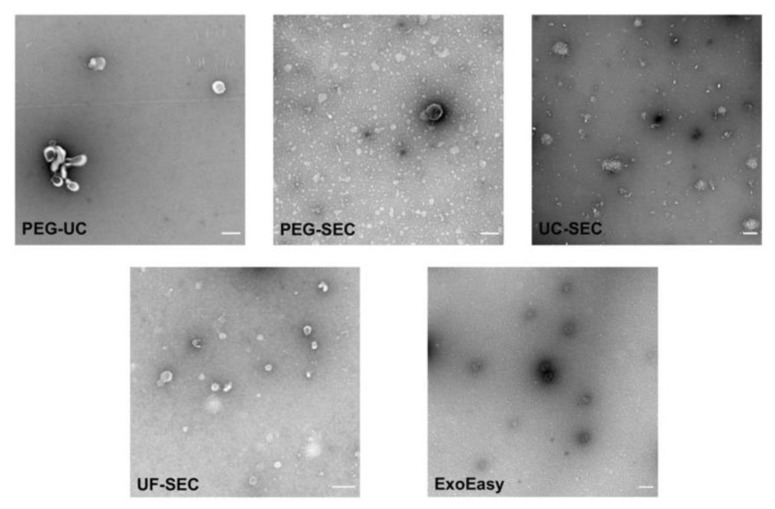
UF-SEC preparation allows the recovery of uEVs with cup-shaped appearance. Representative images of transmission electron microscopic analysis of uEV samples obtained with the different methods as indicated. Size analyses were performed using the ImageJ software. Mean diameters of the EV-like objects were calculated between 67.97 nm (UF-SEC) and 94.73 nm (PEG-UC). Scale bar: 0.2 µm.

To assess the morphology of uEVs that had been prepared with the different methods, TEM analyses were performed (Figure 7). sEV-sized objects were detected in all preparations. Consistent to the IFCM and WB results, the lowest number of EV-sized objects was found in PEG-UC samples. In samples that were prepared by UC, either with the PEG-UC or the UC-SEC method, EV-sized objects were frequently aggregated. Samples that were prepared with the ExoEasy or the UF-SEC method contained higher numbers of non-aggregated EV-sized objects. In UF-SEC samples, these objects showed the EV-typical cup-shaped morphology. Thus, results of the TEM analyses substantiated the IFCM and WB data, indicating UF-SEC apparently allows more efficient preparation of uEVs compared to the other methods tested here.

## 3. Discussion

Urine is an ideal source of biomarkers due to its non-invasive collection. Apparently, uEVs reflect physiological processes within the kidneys and the urinary tract and are therefore increasingly considered to provide a novel class of urinary biomarkers for the diagnosis and risk stratification of kidney diseases [4,35]. However, reflecting the situation in the whole EV field, EV enrichment and detection strategies are still not entirely optimized. The efficiency of EV preparation protocols and the reliability of EV analysis devices remain under discussion, certainly aggravating translation of EV-based biomarkers into clinical routine. The new position paper from the ISEV urinary EV task force is an important milestone in standardization of uEV research and has enabled a perspective on poorly studied parameters in uEV experiments [24], of which some of the most urgent were investigated in this manuscript. Here, upon applying reported uEV isolation strategies, we prepared uEVs from void urine of healthy donors and compared obtained samples using different analysis technologies. As an important finding of our study, we reported incongruencies of NTA and IFCM data. While the numbers of CD9^+^ objects measured by IFCM correlated with WB intensities of TSG101 bands but not with those of UMOD, the particle numbers recorded in NTA also correlated with UMOD amounts.

The highest recoveries of CD9^+^ objects were found in UF-SEC samples. In contrast, the highest particle numbers were recovered in ExoEasy samples. Supporting the CD9-IFCM data, UF-SEC samples exposed the highest contrast between the TSG101 and UMOD bands in Western Blots and contained the highest number of cup-shaped vesicle-like objects. Thus, we concluded that among the methods tested here, UF-SEC provides the best method for uEV preparation.

UF-SEC was introduced as a feasible uEV preparation method in 2015 [36]. Until now, only very few reports have been published that comprehensively compared the accuracy of UF-SEC to other uEV preparation methods applied in the field. A recent study, which was conducted in parallel to our investigations, compared the efficiency of four EV preparation methods [23]. Although the authors used NanoFCM in parallel to classical NTA, they focused their conclusions mainly on particle numbers and purities assessed as particle per protein ratio. Upon comparing UF-SEC, UC, polymer-based precipitation using a commercial reagent and Exodisc microfluidics for the preparation of uEVs, the authors identified Exodisc as the best method, followed by UF-SEC [23]. However, according to WB, the highest EV marker intensities were apparently recovered in the UF-SEC sample. In good agreement with our findings, many fewer EV marker proteins were recovered in the UC sample and no EV marker protein in the precipitation sample, although especially UC—combined with SEC—had been described as a feasible method for uEV preparation before [15,19]. In contrast to the procedure we used for IFCM analyses, the authors had to remove unbound antibodies for the NanoFCM analyses by a subsequent washing and UC-based precipitation step. Thus, quantitative analysis was not achievable. Since we and others indeed observed significant EV loss during UC [28], we share the authors’ view that quantitative analysis following a UC-based washing procedure needs to be performed with care. However, similar to the results of our study, we would carefully like to conclude from their WB results that also in their hands UF-SEC appeared as the most favorable method tested. Notably, with the NanoFCM in good agreement to our IFCM data, the authors detected many more CD9^+^ than CD63^+^ and CD81^+^ objects in their uEV samples [23], highlighting CD9 as an important uEV marker protein.

Based on small particle recovery, a previous study reported that the highest small particle numbers (50–150 nm) were recovered in samples that were prepared with the UC-SEC method [19]; they contained slightly more small particles than samples prepared using UF-SEC. Consistent to our study and that of Dong and colleagues [23], PEG and PEG-SEC processing resulted in low small particle yields [19]. In terms of purity, UC samples contained many more proteins than UC-SEC and UF-SEC samples. Since UC-SEC preparations contained more small particles than UF-SEC preparations, the authors identified UC-SEC as the best among their tested methods [19]. However, despite the information that exosomal marker protein contents were below the detection limits in PEG and PEG-SEC samples but detectable in the other samples, WB analyses were not presented.

In agreement with our results, polymer or PEG precipitation, which we previously qualified as a very reproducible method for EV preparation from animal sera or conditioned cell culture media [31], is obviously not appropriate for the preparation of urinary EVs [19,23]. Efficacies of PEG precipitation procedures depend on several parameters including pH, salt, and protein concentrations [37,38], which are apparently not in the permissive range in void urine samples.

Our data demonstrated that the interpretation of method comparisons largely depends on the selection of the EV characterization strategies and the weighting of the results. Underestimating the presence of small non-vesicular particles in classical EV preparations, we and others have introduced NTA as an “exosome” characterization and quantification device [25,26], which was quickly adopted by the field. Until today, particle quantification, most frequently performed by NTA, was considered to be an essential part of experiments fulfilling the minimal information for studies of extracellular vesicles (MISEV) criteria [7]. However, although the issue of non-vesicular particle contamination is increasingly noticed [39], uncritical interpretation of particle counts may have severely influenced the choice of EV preparation methods. The side-by-side comparison performed here clearly demonstrated the limitations of traditional NTA in EV purification comparisons. Although we have focused our IFCM analyses on the detection of CD9, this approach showed a correlation to EV-associated proteins detected by WB. In fact, we found that CD9 is the most abundant EV marker out of the three classically used tetraspanins, CD9, CD63, and CD81, in human urine, which is also supported by a recent report of another group using NanoFCM flow cytometry [23]. As it was previously reported that the excretion of CD9-positive vesicles strongly correlates with urinary creatinine, the most frequently applied clinical marker for urine volume normalization [40], we would conclude from our data that CD9 is a suitable surrogate marker for the estimation of uEV abundancy, even though formally it may not cover all subpopulations. Of note, this is also the case for NTA, which fails to detect highly abundant urinary EVs smaller than 70 nm [41]. Since NTA data correlated with the UMOD content in the obtained samples, our data implied that at least a proportion of co-prepared UMOD was detected as particles within comparable size ranges as those of sEVs, as was already reported for IgG immunoglobulins, myosin aggregates, and alpha-synuclein [42,43]. In a related manner, McNicholas and colleagues previously reported that NTA analyses of uEV preparations from macroalbuminuric disease patients are severely confounded by the presence of albumin. Furthermore, as in our study, their NTA and WB data were discrepant [30].

Apart from questioning the reliability of NTA in its traditional form for the comparison of EV preparation methods, our data revealed that care should also be taken when storage conditions for EV-containing biofluids are explored. Given the ability of IFCM to detect stained uEVs in unprocessed samples, we observed a severe impact of the storage condition of preprocessed void urine samples on CD9^+^ uEV recovery. The lowest loss of uEVs occurred when samples were stored at −80 °C and the highest—more than 90%—when they were stored at −20 °C. Notably, these findings correspond well to WB results of a previous study, which also investigated impacts of different urine storage temperatures [44]. In contrast, NTA analyses of void urine samples failed to detect any storage temperature-dependent particle losses [45].

As confirmed by our data, methods with the highest *bona fide* uEV recovery may not yield the highest particle concentrations. Accordingly, we would like to recommend the re-evaluation of former method comparisons, especially when conclusions were mainly based on particle recoveries. This also demonstrates the urgent need for next-generation EV analysis technologies and devices. For instance, advanced NTA devices have been developed, which allow specific tracking of fluorescently labeled particles. Tracking results, however, depend on the labeling efficacies of fluorescent dyes or fluorochrome-labeled antibodies, which both come along with their own challenges [46]. In addition to advanced NTA and IFCM, flow cytometers have been developed to efficiently record single objects in size range of sEVs, such as the NanoFCM device [47]. Based on plasmon resonance, the NanoView device can also analyze the presence of sEVs at the single object level [48]. Moreover, a novel direct stochastic optical reconstruction (dSTORM) device, the Nanoimager, also allows single sEV analyses [49]. In summary, all of these second-generation techniques have their own strengths and weaknesses, but certainly will help to optimize EV preparation protocols and increase our overall understanding of EV biology.

## 4. Materials and Methods

### 4.1. Urine Sample Collection and General Preparation

Void urine samples were collected from healthy Caucasian adult male and female volunteers following obtained oral and written informed consent. The study was approved by the ethics committee of the Medical Faculty of the University of Duisburg-Essen (no. 18-8494-BU). Random spot urine was collected in sterile, leak-proof plastic containers (Sarstedt, Nümbrecht, Germany) and the absence of leukocytes, nitrite-producing bacteria, blood, or protein was confirmed by a urinary dipstick test (Multistix 10 SG, Siemens Healthineers, Erlangen, Germany). After collection, the samples were immediately processed. They were first centrifuged at 500× *g* for 10 min (4 °C) and 3000× *g* for 20 min (4 °C) to remove cells, cellular debris, and larger vesicles. Supernatants were stored at −80 °C in 10 mL aliquots in sterile conical tubes (Falcon, Corning, NY, USA) until further processing unless otherwise indicated. Details of the centrifugation procedures are provided in Supplementary Table S4.

Urine samples considered for direct IFCM analyses without previous sEV enrichment were pre-cleared by filtration before antibody staining using 0.22-µm polyethersulfone syringe filters (Minisart, Sartorius, Göttingen, Germany).

### 4.2. Polyethylene Glycol Precipitation Followed by Ultracentrifugation (PEG-UC)

PEG precipitation and subsequent UC was performed as described previously [31], with some minor modifications. Briefly, pre-processed urine samples were centrifuged for 45 min at 10,000× *g* and 4 °C. Following addition of 1 mL 50 *w/v* % PEG-6000 (Sigma-Aldrich, Steinheim, Germany) and 750 µL of 0.9% NaCl (Fresenius Kabi, Bad Homburg, Germany) to 8.25 mL supernatant (final PEG concentration: 10%), uEVs were precipitated at 4 °C overnight. Precipitates were pelleted by centrifugation for 30 min at 1500× *g* and 4 °C. Pellets were resuspended in 0.9% NaCl and centrifuged at 110,000× *g* for 2 h at 4 °C in an XPN-80 ultracentrifuge equipped with a Type 50.4 Ti rotor (Beckman Coulter, Krefeld, Germany; k-factor: 93). UC pellets were resuspended in 1 mL of 0.9% NaCl and stored at −80 °C until further analysis.

### 4.3. PEG Precipitation Followed by SEC (PEG-SEC)

Urine samples were pre-processed, as indicated above. Then, 200 µL of PBS (ThermoFisher Scientific/Gibco, Carlsbad, CA, USA) were added to 7.8 mL pre-processed urine. Following addition of 2 mL 50% *w*/*v* PEG-6000 solution (Sigma-Aldrich; final concentration: 20%), uEVs were precipitated at 4 °C overnight. Precipitates were pelleted by centrifugation for 35 min at 1500× *g* and 4 °C. Next, size exclusion chromatography was performed with 10 mL of Sepharose CL-2B (GE Healthcare, Uppsala, Sweden) using self-packed, PBS-equilibrated columns (Econo-Pac 20 mL, Bio-Rad Laboratories, Hercules, CA, USA), according to the “Mini-SEC” procedure reported by Hong and colleagues [50]. The resuspended uEV pellet was applied onto the top mesh of the size exclusion columns. Six fractions, each of 1 mL, were eluted. After elution of each fraction, 1 mL PBS was added to the column. Fraction 4, the fraction containing most EVs, was used for all downstream analyses.

### 4.4. UC Followed by Size Exclusion Chromatography (UC-SEC)

For the UC-SEC-method, pre-processed urine samples were centrifuged for 45 min at 17,000× *g* at 4 °C. Next, supernatants were centrifuged for 70 min at 200,000× *g* and 4 °C in an XPN-80 ultracentrifuge equipped with a Type 50.4 Ti rotor (Beckman Coulter, Krefeld, Germany; k-factor: 51). Obtained pellets were resuspended and washed in 2 mL PBS, followed by pelleting uEVs again by repeating the UC step. SEC of resuspended pellets was performed as described above.

### 4.5. Ultrafiltration Followed by SEC (UF-SEC)

UF-SEC was performed according to the protocol by Monguió-Tortajada et al. [32] with some minor modifications. Ten mL of pre-processed urine was centrifuged at 17,000× *g* for 15 min at 4 °C. The supernatant was then supplemented with 5 mL of 0.9% NaCl and concentrated by centrifugation (4000× *g*; 10 min; room temperature) using Amicon Ultra-15,100 kDa centrifugal filtration units (regenerated cellulose; Merck/Millipore, Cork, Ireland). To further reduce the protein content, the concentrate above the filter was diluted with 0.9% NaCl to a final volume of 10 mL and re-concentrated by centrifugation. Concentrates were harvested and supplemented with 0.9% NaCl to a final volume of 1 mL. Thereafter, SEC was performed as described above.

### 4.6. Ultrafiltration Followed by Immunoaffinity Capturing (UF-MACS)

Immunoaffinity isolation of uEVs was performed using a commercially available kit (Exosome Isolation Kit Pan, human, Miltenyi Biotec, Bergisch Gladbach, Germany), according to the manufacturer’s instructions. The kit is based on the immunomagnetic isolation of uEVs carrying any of the surface epitopes CD9, CD63, or CD81. Briefly, pre-processed void urine was pre-cleared by centrifugation at 10.000× *g* for 30 min (4 °C). Then, 10 mL of the supernatant were concentrated using an Amicon Ultra-15 100 kDa filter (regenerated cellulose; Merck/Millipore) by centrifuging at 4.000× *g* for 10 min. The concentrate was adjusted to 2 mL with 0.9% NaCl. Then, 50 µL of antibody-loaded magnetic beads were added. After incubation for 1 h at room temperature, the sample was loaded onto an equilibrated separation column within a magnetic stand (MACS MultiStand with µMACS separator, Miltenyi Biotec). After serial washing steps, the column was removed from the magnetic stand. Then, uEV-bead aggregates were eluted by flushing with 100 µL of the supplied isolation buffer. The samples were adjusted to 1 mL with 0.9% NaCl.

### 4.7. Membrane Affinity-Based Isolation (ExoEasy)

The commercial ExoEasy Maxi Kit (Qiagen, Hilden, Germany) was used for membrane affinity-based isolation of uEVs, according to the instructions of the manufacturer. Briefly, pre-cleared void urine was centrifuged at 10.000× *g* for 45 min (4 °C). The supernatant was mixed in a 1:1 ratio with the provided binding buffer and applied to the provided centrifugation columns. After centrifugation, membranes were washed, and bound components were eluted by the addition of 1 mL of the provided elution buffer and subsequent centrifugation.

### 4.8. Imaging Flow Cytometry (IFCM)

IFCM was performed on the AMNIS ImageStreamX Mark II Flow Cytometer (AMNIS/Luminex, Seattle, WA, USA), as described before [27,29]. Details for all antibodies used are provided in Supplementary Table S5. Generally, antibody incubation was performed for 1 h at room temperature. According to the recommendations of the MIFlowCyt-EV guidelines [47], unstained uEV samples, NaCl-HEPES buffer with antibodies but without uEV sample, as well as stained samples supplemented with 1% NP40 (Calbiochem, San Diego, CA, USA) were analyzed as controls (Supplementary Figure S6). After staining, samples were diluted with PBS and analyzed using the built-in autosampler for 96-well, round-bottom plates. Acquisition time was selected to be 5 min per well. Data were acquired at 60× magnification, low flow rate and with removed beads option deactivated. Further details are provided in Supplementary Tables S7 and S8.

Data were analyzed as described previously using the IDEAS software (version 6.2) [27,29]. Fluorescent events were plotted against the side scatter (SSC). A combined mask feature was used (MC and NMC) to improve the detection of fluorescent images. Images were analyzed for coincidences (swarm detection) by using the spot counting feature. Every data point with multiple objects was excluded from the analyses. Events with low side scatter values (<500) and fluorescence intensities higher than 300 were considered as uEVs. Average concentrations were calculated according to the acquisition volume and time.

### 4.9. Nanoparticle Tracking Analysis (NTA)

Average size distribution and particle concentration analyses of uEV samples were performed by NTA on the ZetaView PMX-120 platform equipped with the software version 8.03.08.02 (ParticleMetrix, Meerbusch, Germany), as described before [31]. Briefly, samples were diluted in NaCl. One mL diluted sample volume was loaded into the flow cell and recorded for 55 s. Particle sizes and numbers of all 11 positions were recorded and calculated as the mean of the results. The following settings were used: positions: 11; cycles: 5; quality: medium; min. brightness: 20; min. size: 5; max. size: 200; trace length: 15; sensitivity: 75; shutter: 75; and framerate: 30.

### 4.10. SDS-PAGE/Immunoblot

Equal volumes of each sample were lysed in radioimmunoprecipitation assay buffer (1:1; 150 mM NaCl, 50 mM Tris, 1% Triton X-100, 0.1% SDS, 0.5% sodium desoxycholate) containing protease inhibitors (1:100; Pefabloc SC, Sigma-Aldrich). Proteins were separated under reducing and non-reducing conditions on Mini-Protean TGX Any-kD precast gels (Bio-Rad, Hercules, CA, USA) and transferred to nitrocellulose membranes (GE/Amersham, Buckinghamshire, UK) using a Fastblot B34 blotting device (Biometra, Göttingen, Germany). Membranes were blocked with 5% milk powder (Sigma-Aldrich, St. Louis, MO, USA) solved in PBS-T. Membranes were incubated with primary antibodies (Supplementary Table S5) overnight at 4 °C. Following serial washes, bound antibodies were counterstained with horseradish peroxidase-conjugated secondary antibodies (Supplementary Table S5) for 1 h at room temperature. After washing, the Super Signal West Femto Chemiluminescent Substrate (Thermo Scientific, Rockford, IL, USA) was applied according to the manufacturer’s instructions. Obtained signals were documented with the Fusion FX7 detection system (Vilber Lourmat, Eberhardzell, Germany).

### 4.11. Transmission Electron Microscopy (TEM)

A carbon-coated formvar film supported by a 200-mesh copper grid (Plano, Wetzlar, Germany) was pretreated with a glow discharging agent (Ted Pella, Redding, CA, USA) to create a hydrophilic surface. Five µL of each given sample suspension were placed on a grid and incubated for 5 min. The grids were washed three times for 1 min by placing it on 30-µL droplets of deionized water before they were stained for 1 min on a 20-µL droplet of 1.5% aqueous phosphotungstic acid solution (*w*/*v*, Carl Roth, Karlsruhe, Germany). Thereafter, following removal of excess staining solution with a piece of filter paper, they were dried at ambient air. Images were acquired using a JEOL JEM 1400Plus (JEOL, Tokyo, Japan), operating at 120 kV and equipped with a 4096 × 4096 px CMOS camera (TVIPS, Gauting, Germany). The image acquisition software EMMENU (Version 4.09.83) was used for taking 16-bit images. ImageJ software (Version 1.52b, https://imagej.nih.gov/ij/, accessed on 1 October 2021) was used to process and analyze obtained images.

### 4.12. Data Analysis and Statistics

Computational data plotting, analysis, and visualization was performed using Microsoft Excel 2019 and GraphPad Prism (version 8.4.0). Image analysis, including band densitometry, was performed using ImageJ. Band density was calculated by measuring the density of a region of interest (ROI) placed around the bands after subtraction of background density. 

All measurement results are given as means ± standard deviation, unless otherwise indicated. The normal distribution of data was tested using the Anderson–Darling test for ≥8 individual values and the Shapiro–Wilk test for ≤7 individual values. Statistical significance of comparisons was calculated using the unpaired Student’s *t*-test or, in the case of more than two groups, a one-way ANOVA with Tukey’s post-test. Multiple comparisons of non-parametric data were performed using the Kruskal–Wallis test with Dunn’s post-test. Data correlation analysis of non-parametric datasets was performed using Spearman’s correlation coefficient with a two-tailed Student’s *t*-test. *p* values < 0.05 were considered as significant. Levels of significance are indicated as follows: *p* ≤ 0.05 (*); *p* ≤ 0.01 (**); *p* ≤ 0.001 (***); *p* ≤ 0.0001 (****). 

## 5. Conclusions

The EV field is progressing almost exponentially and provides plenty of novel diagnostic and therapeutic opportunities. Furthermore, EV studies will certainly help to increase the basic understanding of physiological and pathophysiological processes. Despite the increasing amount of knowledge in the recent decade, the methods for preparing and characterizing EVs remain to be optimized. As demonstrated here, the results generated by next-generation analysis devices may question interpretations of past findings and may help to identify experimental weaknesses in current EV preparation technologies. Upon recognizing existing pitfalls and limitations, new technical challenges will arise and help to evolve the field more accurately. Here, we used IFCM to identify UF-SEC as a powerful method to enrich uEVs from void urine.

## Data Availability

We have submitted all relevant data of our experiments to the EV-TRACK knowledgebase (https://evtrack.org, accessed on 1 October 2021); EV-TRACK ID: EV210067) [51].

## Supplementary Materials

The following are available online at https://www.mdpi.com/article/10.3390/ijms222212436/s1.
