# Supplementary material for: Single Extracellular Vesicle Analysis Performed by Imaging Flow Cytometry and Nanoparticle Tracking Analysis Evaluate the Accuracy of Urinary Extracellular Vesicle Preparation Techniques Differently
Source: Int J Mol Sci. 2021 Nov 18;22(22):12436. doi: 10.3390/ijms222212436 (PMC8620260; doi:10.3390/ijms222212436)
Supplement: Supplementary file 1 [file ijms-22-12436-s001.zip › ijms-1425540-supplementary.pdf]

Supplementary Material

Single Extracellular Vesicle Analysis Performed by Imaging Flow Cytometry and Nanoparticle Tracking Analysis Evaluate the Accuracy of Urinary Extracellular Vesicle Preparation Techniques Differently

Marvin Droste [1], Tobias Tertel [2], Stefanie Jeruschke [1], Robin Dittrich [2], Evangelia Kontopoulou [3], Bernd Walkenfort [4], Verena Börger [2], Peter F. Hoyer [1], Anja K. Büscher [1,*,†], Basant K. Thakur [3,*,†] and Bernd Giebel [2,*,†]

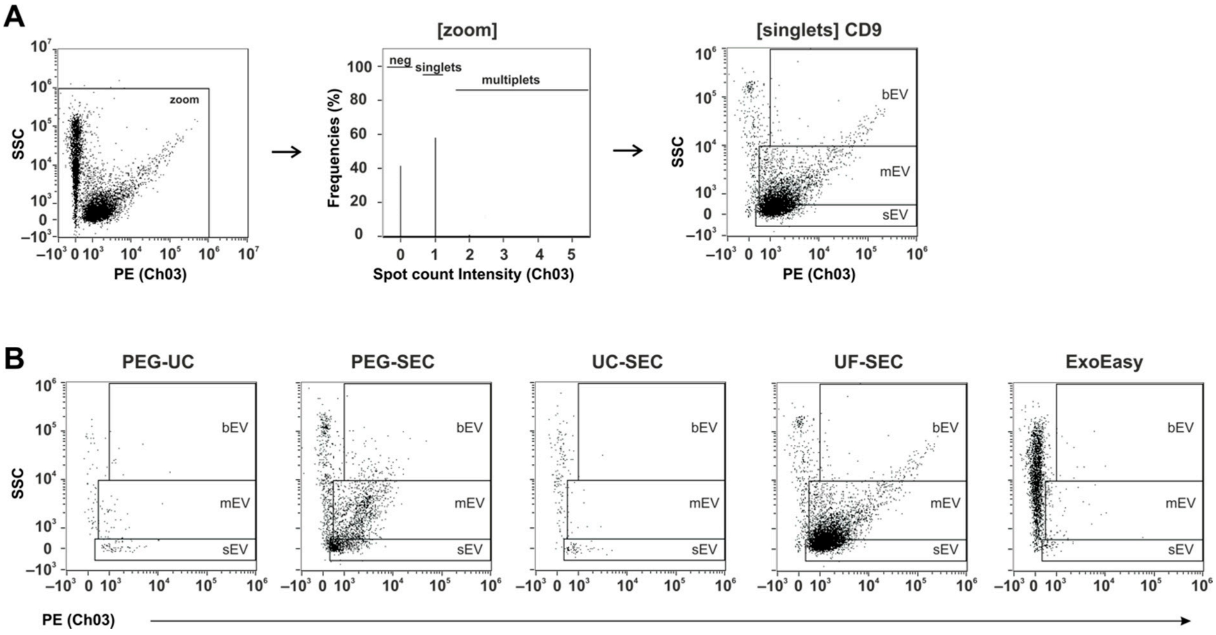

Supplementary Figure S1. (A) Gating strategy for the detection of CD9⁺ objects using IFCM. At first, all recorded objects were plotted based on their side scatter signals (SSC) and their fluorescence intensities following anti-CD9 antibody staining (1st plot). For downstream analyses, coincidences (swarm detection) and objects lacking any fluorescent signal were discriminated from single fluorescent objects (singlets). Only singlets were considered in all downstream analyses. Singlets were also plotted in SSC to fluorescence intensity diagrams. Based on the SSC intensity, three different object subgroups were discriminated: small EVs (sEV), medium-sized EVs (mEV) and big EVs (bEV). (**B)** Representative flow plots of uEV samples prepared with all five methods.

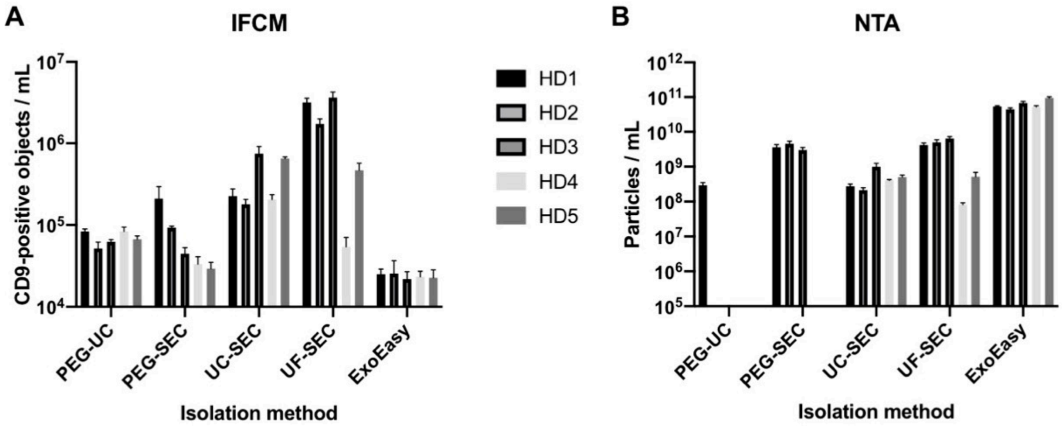

Supplementary Figure S2. Detail of Fig. 4: IFCM and NTA results of all prepared uEV samples measured by IFCM (**A**) or NTA (**B**), respectively. Error bars in **A** represent the standard deviation of three independent IFCM analyses of each sample and those in **B** the standard deviation of the data measured at 11 camera positions per sample using NTA.

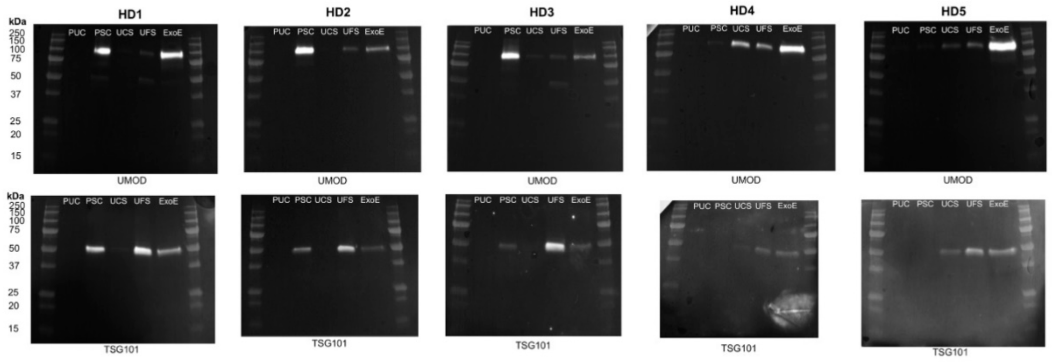

Supplementary Figure S3. Images of the TSG101 and UMOD Western blots of the uEV preparations obtained from all void urine samples with the different applied methods. Sample loading of -80°C stored uEV samples was adjusted to volume equivalents of the initial void urine sample. Following sample separation under reducing conditions membranes were sequentially probed with anti-TSG101 and anti-UMOD antibodies (without stripping). HD = healthy donor sample, PUC: PEG-UC; PSC: PEG-SEC; UCS: UC-SEC; UFS: UF-SEC; ExoE: ExoEasy.

Supplementary Table S4. Data about the centrifuges, rotors and the centrifugation speed. SW = swing-out rotor, FA = fixed-angle rotor.

Speed	Centrifuge	Manufacturer	Rotor Name	Rotor Type
≤ 4000x *g*	Heraeus Megafuge 16R	ThermoFisher Scientific, Osterode, Germany	TX-400	SW
10,000x *g*<br>17,000x *g*	Sorvall RC6+	ThermoFisher Scientific, Osterode, Germany	HB-6	SW
> 100,000x *g*	Optima XPN-80	Beckman Coulter, Krefeld, Germany	Type 50.4 Ti	FA

Supplementary Table S5. List of the applied antibodies and isotype controls including information about their clone and ordering number as well as the applied dilution factor. *The CD9 antibody (clone VJ1/20) was kindly provided by Dr. F. Sánchez-Madrid, Madrid, Spain.

Antibody	Clone, cat. #	Application	Manufacturer	Dilution
CD9-PE (mouse)	MEM-61, 1P-208-T100	IFCM	Exbio, Vestec, Czech Republic	1:100
CD9 (mouse)	VJ1/20	WB	F. Sánchez-Madrid*	1:1,000
CD63-APC (mouse)	MEM-259, 1A-343-T100	IFCM	Exbio, Vestec, Czech Republic	1:100
CD81-FITC (mouse)	JS-64, B25329	IFCM	Beckman Coulter, Indianapolis, IN, USA	1:100
TSG101 (rabbit)	Polyclonal HPA006161	WB	Atlas Antibodies, Bromma, Sweden	1:1,000
THP (UMOD)	B-2 sc-271022	WB	Santa Cruz, Dallas, TX, USA	1:200
Goat anti-rabbit IgG-HRP	Polyclonal sc-2004	WB	Santa Cruz, Dallas, TX, USA	1:10,000
Rabbit anti-mouse IgG-HRP	Polyclonal sc-358914	WB	Santa Cruz, Dallas, TX, USA	1:10,000
Mouse IgG2a-FITC	S43.10 130-113-833	IFCM	Miltenyi Biotec, Bergisch Gladbach, Germany	1:250
Mouse IgG1-PE	MOPC-21 555749	IFCM	BD Biosciences, Heidelberg, Germany	1:250
Mouse IgG1-APC	MOPC-21 400122	IFCM	BioLegend, San Diego, CA, USA	1:250

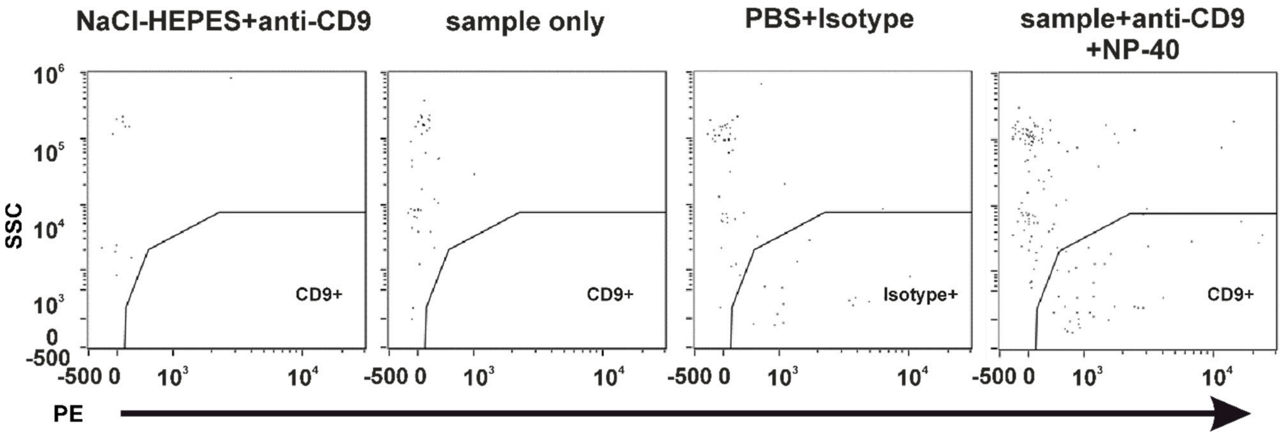

Supplementary Figure S6. Experimental controls in accordance with the MIFlowCyt-EV framework.

Supplementary Table S7. Applied laser settings for imaging flow cytometry.

Laser [nm]	Used Power [mW]	Max. Power [mW]	Filter [nm]
375	70	70	–
488	100	100	FITC (Ch02) 480-560
561	200	200	PE (Ch03) 560-595
648	150	150	APC (Ch11) 642-745
785 (SSC)	70	70	SSC (Ch06) 756-780

Supplementary Table S8. Applied compensation matrix for imaging flow cytometry.

	Ch1	Ch2	Ch3	Ch4	Ch5	Ch6	Ch7	Ch8	Ch9	Ch10	Ch11	Ch12
**Ch1**	1	0.029	0.042	0	0	0	0	0	0	0	0.002	0
**Ch2**	0.051	1	0.05	0	0	0	0	0	0	0	0.002	0
**Ch3**	0	0.13	1	0	0	0	0	0	0.02	0	0.002	0
**Ch4**	0	0.064	0.49	1	0	0	0	0	0	0	0.003	0
**Ch5**	0	0.017	0.155	0	1	0	0	0	0	0	0.074	0
**Ch6**	0.015	0.02	0.04	0	0	1	0	0	0	0	0.01	0
**Ch7**	0.023	0.003	0.003	0	0	0	1	0	0.015	0	0.024	0
**Ch8**	0	0.032	0.008	0	0	0	0	1	0.012	0	0.023	0
**Ch9**	0	0.004	0.084	0	0	0	0	0	1	0	0.024	0
**Ch10**	0	0.002	0.041	0	0	0	0	0	0.084	1	0.028	0
**Ch11**	0	0.001	0.012	0	0	0	0	0	0.025	0	1	0
**Ch12**	0	0	0.003	0	0	0	0	0	0.013	0	0.125	1
